# Development of Plate Reader and On-Line Microfluidic Screening to Identify Ligands of the 5-Hydroxytryptamine Binding Protein in Venoms

**DOI:** 10.3390/toxins7072336

**Published:** 2015-06-24

**Authors:** Reka A. Otvos, Janaki Krishnamoorthy Iyer, René van Elk, Chris Ulens, Wilfried M. A. Niessen, Govert W. Somsen, R. Manjunatha Kini, August B. Smit, Jeroen Kool

**Affiliations:** 1 AIMMS Division of BioAnalytical Chemistry, Faculty of Sciences, VU University Amsterdam, De Boelelaan 1083, 1081 HV Amsterdam, The Netherlands; E-Mails: r.a.otvos@vu.nl (R.A.O.); w.m.a.niessen@vu.nl (W.M.A.N.); g.w.somsen@vu.nl (G.W.S.); 2 Department of Molecular and Cellular Neurobiology, Center for Neurogenomics and Cognitive Research, Neuroscience Campus Amsterdam, VU University Amsterdam, De Boelelaan 1085, 1081 HV Amsterdam, The Netherlands; E-Mails: rene.van.elk@vu.nl (R.E.); guus.smit@vu.nl (A.B.S.); 3 Department of Biological Sciences, Faculty of Science, National University of Singapore, 14 Science Drive 4, Singapore 117543, Singapore; E-Mails: janaki@u.nus.edu (J.K.I.); dbskinim@nus.edu.sg (R.M.K.); 4 Laboratory of Structural Neurobiology, Department of Cellular and Molecular Medicine, Katholieke Universiteit Leuven, Herestraat 49, PB 601, B-3000 Leuven, Belgium; E-Mail: chris.ulens@med.kuleuven.be; 5 Hyphen MassSpec, de Wetstraat 8, 2332 XT Leiden, The Netherlands

**Keywords:** 5-HT_3_ receptor, 5HTBP, high-resolution screening, snake venoms, nano-LC-MS

## Abstract

The 5-HT_3_ receptor is a ligand-gated ion channel, which is expressed in the nervous system. Its antagonists are used clinically for treatment of postoperative- and radiotherapy-induced emesis and irritable bowel syndrome. In order to better understand the structure and function of the 5-HT_3_ receptor, and to allow for compound screening at this receptor, recently a serotonin binding protein (5HTBP) was engineered with the Acetylcholine Binding Protein as template. In this study, a fluorescence enhancement assay for 5HTBP ligands was developed in plate-reader format and subsequently used in an on-line microfluidic format. Both assay types were validated using an existing radioligand binding assay. The on-line microfluidic assay was coupled to HPLC via a post-column split which allowed parallel coupling to a mass spectrometer to collect MS data. This high-resolution screening (HRS) system is well suitable for compound mixture analysis. As a proof of principle, the venoms of *Dendroapsis polylepis*, *Pseudonaja affinis* and *Pseudonaja inframacula* snakes were screened and the accurate masses of the found bioactives were established. To demonstrate the subsequent workflow towards structural identification of bioactive proteins and peptides, the partial amino acid sequence of one of the bioactives from the *Pseudonaja affinis* venom was determined using a bottom-up proteomics approach.

## 1. Introduction

5-Hydroxytryptamine (5-HT, serotonin) is a neurotransmitter acting in the peripheral and central nervous systems. In the brain, it is involved in diverse types of functions, such as anxiety responses, learning and memory, sleep, and behavior [1]. There are at least 15 types of 5-HT receptors belonging to the G-protein coupled receptors. In contrast, there is only one type of 5-HT_3_ receptor (5-HT_3_R), consisting of two types of subunits [2], which belongs to the Cys-loop family of pentameric ligand-gated ion channels (pLGIC) [3,4]. Besides 5-HT_3_R_A/B_, the Cys-loop receptor family includes γ-aminobutyric acid A (GABA_A_) receptors, glycine receptors (GlyRs) and nicotinic acetylcholine receptors (nAChRs) [5]. Antagonists of 5-HT_3_R are in use in the clinic as anti-emetics to control chemotherapy-induced and postoperative nausea and vomiting. This receptor is also a validated drug target for irritable bowel syndrome and it is has been suggested to play a role in various brain disorders, such as schizophrenia and anxiety [6,7].

Screening for novel ligands acting on the 5-HT_3_R is usually performed by radioligand binding assays, radioactive ion-flux assays and/or by performing low-throughput electrophysiological patch-clamp studies [8]. Since the 5-HT_3_R is an ion channel, cell-based assays which involve measuring the membrane potential using fluorescence dyes have been developed [9]. These assays provide useful functional information on potential ligands for the receptor.

Recently, a binding protein was engineered which contains the ligand recognition properties of the 5-HT_3_R. This ligand-binding pocket of the 5-HT_3_R was engineered by mutation in the original scaffold of the *Aplysia californica* acetylcholine-binding protein (AChBP) [10]. The AChBP is most similar to the extracellular ligand-binding domain of α7-nAChR [11]. The scaffold of the AChBP was a suitable starting point for engineering the 5-HT_3_-binding protein (5-HTBP) because of the high sequence and structural identity of 5-HT_3_R and α7-nAChR [12]. In this regard, many α7-nAChR ligands, such as varenicline [13] and epibatidine [14], bind to the 5-HT_3_R as well, and 5-HT_3_R antagonist tropisetron is a selective agonist of α7-nAChR [15].

In the case of screening complex mixtures, low-throughput bioassay-guided fractionation techniques [16,17], or newer analytical techniques such as high-resolution screening (HRS) are required [18]. High resolution screening (HRS) is a post-column methodology in which a bioassay is coupled directly on-line with a chromatographic separation. Often, via a post-column split, mass spectrometry (MS) is performed in parallel for the identification of compounds. The first HRS systems were developed by the research groups of Przyjazny [19] and Irth [20]. One of the recent advances in the field of HRS is the development of miniaturized systems in which nano-LC separation is coupled post-column to an on-line microfluidic assay along with parallel MS detection [21] As microfluidic on-line assays use very low sample volumes, such technologies are most suitable when only small quantities of sample are available for the analysis [22,23,24]. The major advantage of direct post-column bioaffinity analysis is the capability of analyzing individual compounds in mixtures (such as venoms) after chromatographic separation. The parallel MS detection provides mass and MS/MS data for the identification of bioactive compounds observed.

Previously, we developed an assay for AChBP ligands in HRS [25] and miniaturized-HRS format [21]. In this study, we took advantage of the homology of AChBP with 5HTBP and developed a fluorescence enhancement based assay for 5HTBP ligands. After optimizing and validating the assay in a 96-well-plate format, it was transferred to a microfluidic on-line HRS format allowing the analysis of individual bioactives in complex mixtures. The system consisted of nano-LC separation with a post-column split allowing parallel 5HTBP assay and MS detection. This microfluidic on-line assay has the added advantages of needing only small quantities of samples and low consumption of assay materials. The potential of the microfluidic on-line HRS was demonstrated by screening venoms of the snakes *Dendroapsis polylepis*, *Pseudonaja affinis* and *Pseudonaja inframacula* for ligands of the 5HTBP.

## 2. Results and Discussion

### 2.1. Selection of a Suitable Tracer Ligand

**Figure 1:**
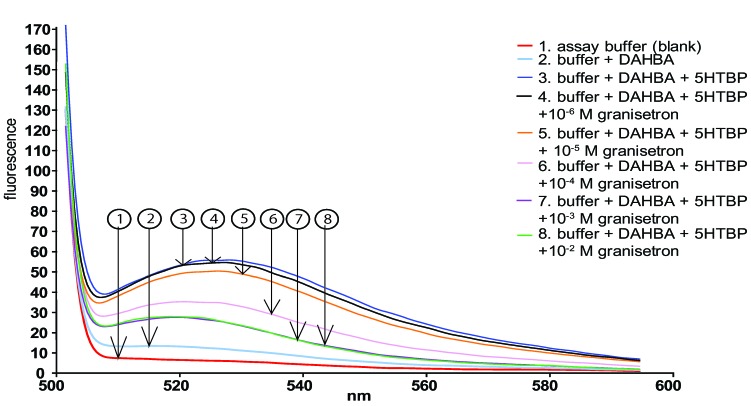
The fluorescence enhancement properties of DAHBA with A1B2D1R 5HTBP. The emission spectrum obtained using excitation at 488 nm is shown between 500 to 595 nm. DAHBA in absence of 5HTBP showed low fluorescence in the 525 nm range (line 2). In presence of D1R 5HTBP the fluorescence was enhanced 4.7 times (line 3). When increasing concentrations (M) of the selective 5-HT_3_R ligand granisetron was added to the mixture, displacement of DAHBA from the 5HTBP binding pocket occurs, resulting in decreased fluorescence intensity (lines 4–8). The concentrations of granisetron in the figure refer to the addition of 10 µL of the indicated concentration to a 1 mL assay mixture in a 1 cm cuvette.

We first evaluated three potentially suitable tracer ligands. These benzylidene anabaseines type tracer ligands were shown to have good fluorescence enhancement properties in the AChBP binding pocket [25]. Since the binding pockets of the AChBP and the 5HTBP are similar (as are the binding pockets of the α7-nAChR and the 5-HT_3_R), as expected, the benzylidene anabaseines showed significant fluorescent enhancement in the presence of the 5HTBP mutant proteins (Figure 1). Fluorescence enhancement factors are defined as fluorescence in presence of 5HTBP divided by fluorescence in absence of 5HTBP. VUF11234, DAHBA and VUF10907 showed fluorescence enhancement factors of 6.5, 4.7 and 3.8, respectively, for the A1B2D1_R_ variant. With the A1B2D1_W_ variant, VUF11234 and VUF10907 showed enhancement factors of 3.7 and 2.5, respectively. Although VUF11234 showed the best fluorescent enhancement with the A1B2D1_R_ mutant, it appeared to undergo notable degradation in solution (deduced by MS analysis). DAHBA was therefore selected as the most suitable tracer ligand. Because the A1B2D1_W_ 5HTBP mutant showed lower fluorescence enhancement with the benzylidene anabaseines, the A1B2D1_R_ mutant was used for the rest of the study.

### 2.2. Fluorescence Enhancement Assay in Microplates

**Figure 2:**
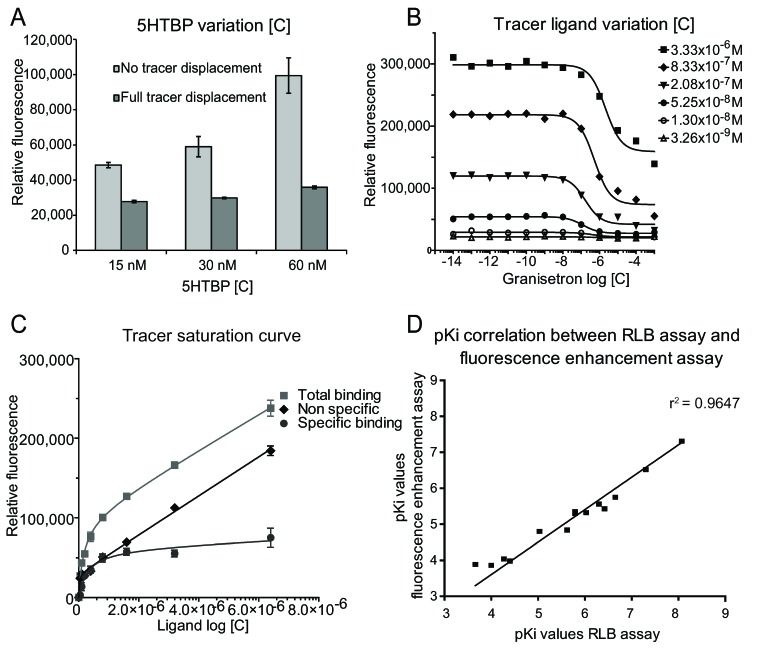
Development of the fluorescence enhancement assay in microplate reader format. (**A**) 5HTBP concentration variation (tracer ligand concentration, 10**^−^**^7^ M). Full tracer displacement was obtained with 100 µM granisetron); (**B**) Optimization of the tracer ligand concentration by establishing IC50 curves of granisetron using different tracer ligand concentrations. The 5HTBP concentration was 50 nM; (**C**) Tracer saturation curves. Specific, non-specific and total binding curves are shown; (**D**) Correlation between the RLB and the microplate reader fluorescence enhancement assay (*r*^2^ = 0.9647). The measured pKi values are in Supplementary Information Table S1.

During assay optimization, the high affinity 5-HT_3_R ligand granisetron was used as competing ligand. In the first experiment, the optimal concentration of 5HTBP was evaluated by comparing no displacement and full tracer displacement at three 5HTBP concentrations (Figure 2A). Increasing concentrations of 5HTBP in the assay evidently enlarged the assay window. The full displacement background signal did not significantly rise upon increasing the 5HTBP concentration. The receptor concentration should be as low as possible in order to allow accurate measurement of high affinity ligands. At the same time, the receptor concentration should be sufficiently high to obtain an adequate assay window. As a compromise, a 5HTBP concentration of 50 nM was chosen, still providing an acceptable assay window (maximum signal-to-noise ratio, S/N) in plate reader format. When very high affinity ligands (K_d_ < 50 nM) have to be measured, a lower binding protein concentration has to be chosen at the expense of a smaller assay window.

The optimal concentration of tracer ligand was determined by generating IC_50_ curves with granisetron using different tracer ligand concentrations (Figure 2B). The optimal concentration of the tracer ligand DAHBA was selected based on an adequate assay window in combination with low background fluorescence. The z’-factors were all similar above a tracer concentration of 5.25 × 10**^−^**^8^ M, but the best S/N (61.1) and signal-to-background ratio (S/B, 3.36) were obtained with 8.33 × 10**^−^**^7^ M tracer concentration (Table 1). This concentration was selected for further plate reader assay optimization and subsequent transfer to the microfluidic on-line assay, providing a z’ score of 0.77.

**Table 1:** Calculated bioassay parameters for different tracer ligand (DAHBA) concentrations.

Tracer c (M)	Z’-factor	Dynamic range	S/B	S/N	SW
3.33 × 10^−6^	0.83	157302	2.16	18.8	4.1
8.33 × 10^−7^	0.77	136610	3.36	61.1	1.5
2.08 × 10^−7^	0.76	76749	3.26	38.0	1.4
5.25 × 10^−8^	0.87	23738	1.84	13.7	9.9
1.3 × 10^−8^	−0.11	7513	1.30	1.8	−4.3
3.26 × 10^−9^	−0.99	3084	1.13	1.01	−5.0
8.14 × 10^−10^	~0	45	1.00	0.01	−5.3

Next, specific and non-specific binding was determined by measuring a tracer saturation curve with and without a displacing ligand. In this experiment, the binding protein concentration was 50 nM while 100 µM granisetron was used as displacing ligand for measurement of the non-specific binding curve. The K_d_ value was then calculated with the use of Prism software and found to be 2.7 × 10**^−^**^7^ ± 2.1 × 10**^−^**^7^ M. A typical saturation curve was obtained, thus showing specific ligand binding to the binding pocket. The non-specific binding, the total binding curve and the resulting specific binding curve is shown (Figure 2C).

The assay was pharmacologically validated by determining the dissociation constants (Ki) of several 5HTBP ligands with the developed fluorescence enhancement assay and comparing these data with Ki values obtained by a radioligand binding (RLB) assay. Fourteen 5-HT_3_R ligands, also binding to the 5HTBP [10], were selected for this experiment. The measured Ki values for both the fluorescence enhancement plate reader assay as well as those measured with the radioligand binding assay are listed (Supplementary Information Table S1). Figure 2D shows the correlation plot of the Ki’s measured with the two assays. The high correlation (*r*^2^ = 0.9647) indicates that the fluorescence enhancement assay is well suited for measuring Ki’s of ligands for the 5HTBP. Furthermore, the affinities measured in both assays are also similar (*i.e.*, they do not all have a same shift to a higher or lower Ki) meaning that similar IC_50_s can be obtained with both assays without the immediate need of calculating Ki’s from IC_50_s.

### 2.3. Microfluidic On-Line Assay Optimization in Nano-LC Flow-injection Analysis Mode

After having optimized the 5HTBP and DAHBA concentrations in microplate reader format and validating and comparing the assay with known ligands, the assay was transferred to the microfluidic on-line assay format. In this format, the non-bound tracer will show a decreased fluorescence when a ligand is displacing the tracer ligand, which will be detected as a negative peak in the assay signal trace. The total signal (height of a negative peak) is measured in flow injection analysis mode, which eliminates any potential interference by the chromatographic column, as the samples directly get into the bioassay allowing testing of the system at different conditions. The concentration of test ligand injected results in full tracer displacement under all conditions to obtain the full bioassay signal under various conditions. The assay window (negative peak height) was increased with higher concentrations of tracer ligand or binding protein in the bioassay. At higher tracer ligand concentrations, there will be increased background fluorescence. In order to minimize the background fluorescence and to avoid using an unnecessary high concentrations of tracer ligand and binding protein, we optimized their concentrations to keep the background fluorescence at the peak minimum.

**Figure 3:**
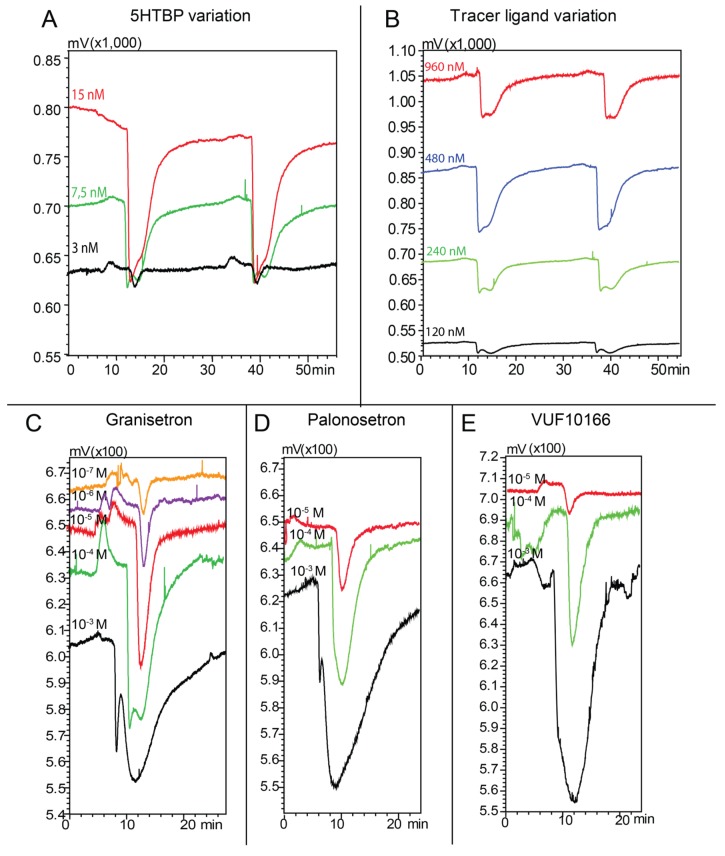
Microfluidic on-line assay optimization using injections of 500 nL 100 µM granisetron. (**A**) Effect of the 5HTBP concentration (3.0, 7.5 and 15 nM) using a tracer concentration of 240 nM; (**B**) Effect of the tracer ligand concentration. Duplicate injections are shown for each condition tested. Negative peaks represent displacement of the fluorescent tracer by the ligand. (**C**–**E**) Serial dilutions of (**C**) granisetron, (**D**) palonosetron and (**E**) VUF10166 in the microfluidic on-line assay. The negative peaks represent the bioactivity of the ligands. When the concentration of a ligand reaches binding pocket saturation, the negative peak height will not further increase and the peak becomes broader due to chromatographic tailing and overloading. The concentrations in Figure (**C**–**E**) refer to the injected concentration of the ligand. The bioassay signals on Figure (**A**,**B**) are non-superimposed, while Figure (**C**–**E**) are showing the signals superimposed.

Firstly, three different concentrations of 5HTBP were tested (3.0, 7.5 and 15 nM; Figure 3A) with a DAHBA concentration of 240 nM, while 500 nL of 100 µM granisetron was injected as competing ligand. Although the 15 nM 5HTBP showed the largest assay window, the baseline fluorescence was close to the highest measurable intensity for the detector. With 7.5 nM 5HTBP, the assay window was lower; however, the baseline fluorescence decreased significantly and the actual S/N was hardly compromised. A 3.0 nM 5HTBP concentration showed a significantly decreased assay window and S/N. From the three concentrations tested (Figure 3A), the 7.5 nM concentration was selected for use in further experiments.

The tracer ligand concentration in the assay mixture was evaluated in the range of 120–960 nM using a 7.5 nM 5HTBP concentration (Figure 3B). Injections of 500 nL of a 100 µM granisetron solution were performed in the microfluidic on-line assay. A 120 nM tracer ligand concentration provided a very small assay window. With a tracer concentration of 240 nM, an acceptable assay window was obtained. Larger assay windows were obtained for tracer concentrations of 480 and 960 nM, but the corresponding background fluorescence signals were too high. Therefore, 240 nM was selected for further use in assay evaluation.

After optimization, the assay was pharmacologically validated by measuring semi-quantitative IC_50_ curves for granisetron, palonosetron, VUF10166, and 5-F-tryptamine (Figure 3C–E). In an on-line format, the concentration actually measured in the assay will be lower than the injected concentration due to addition of the assay mixture and dilution (*i.e.*, band broadening) during the chromatographic process. The dilution due to post-column mixing (D_M_) follows from the ratio of the ultimate flow through the fluorescence detector and the effluent flow of the nano-LC entering the microfluidic incubation chip. The chromatographic dilution (D_C_) depends on the observed full width at half maximum (FWHM) of the compound peak, the flow rate in the nano-LC, and the injection volume. The procedure for calculating the ultimate concentration of the tested compound was described previously [26]. For the present on-line 5HTBP assay, the D_M_ was calculated to be 13.5. The D_C_ was calculated for each chromatographic peak separately. Due to the dilution during on-line screening, it was not possible to measure complete IC_50_ curves, Therefore, the IC_50_ values were completed by adding a data point for the 100% displacement concentration, which was empirically set at 10**^−^**^2^ M. This way, semi-quantitative IC_50_s were used to determine the pKi of granisetron, palonosetron, VUF10166, and 5-F-tryptamine. The resulting pKi values correlated well with the pKi values measured with the RLB assay (*r*^2^ = 0.9598) (Supplementary Information Figure S1).

### 2.4. Microfluidic On-Line HRS of Snake Venoms

After the microfluidic on-line assay was optimized using nano-LC flow injection analysis, a high-resolution mass spectrometer was coupled to the nano-LC system in parallel to the microfluidic on-line assay. With this microfluidic on-line HRS system, several snake venoms were screened for bioactive peptides and proteins. The 5-HT_3_R ligand palonosetron was analyzed every measurement day to determine the proper functioning of the system and also to establish an accurate time alignment of the bioactivity chromatogram obtained with fluorescence detection and the MS chromatogram. Spiking an internal standard in the venom samples was not preferred because none of the available 5-HT_3_ ligands was eluting in the dead-volume time and therefore potentially would interfere with the detection of active co-eluting venom components.

With the microfluidic on-line HRS system, we screened the venoms of the following snakes: *Pseudonaja inframacula*, *Pseudonaja affinis*, *Dendroaspis polylepis*, *Crotalus Adamanteus*, *Crotalus horridus atricaudatus*, *Bitis arietans* and *Bitis nasicornis*. Only the venoms from *Pseudonaja inframacula*, *Pseudonaja affinis* and *Dendroaspis polylepis* snakes were found to contain bioactive peptides that interact with 5HBP. It had been reported that the *Pseudonaja textilis* venom contains neurotoxins (pseudonajatoxin a and b, textilotoxin) which are known to have affinity to the neuronal nAChRs [27]. Their affinity to the 5-HT_3_ receptor, however, has not been established so far, but considering the similarities between the aforementioned receptors it was expected that active binders would be found.

**Figure 4:**
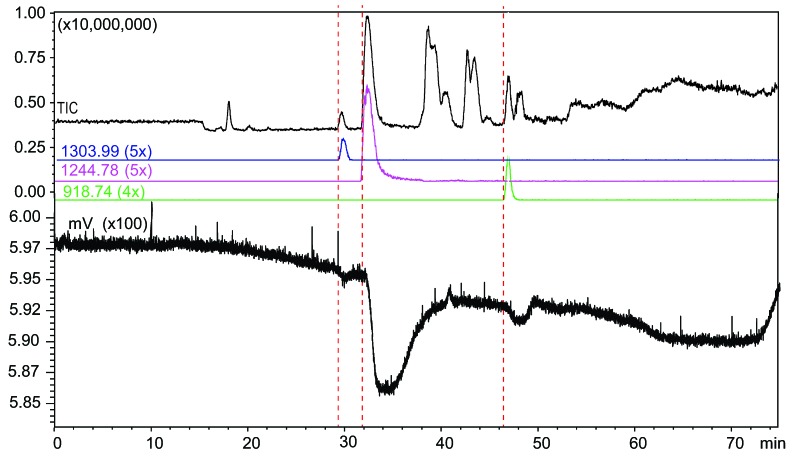
Microfluidic on-line high-resolution screening (HRS) of the venom of *Pseudonaja affinis.* The identified ligands: 32.3 min, *m*/*z* value of 1244.779 (5+ charges, peptide mass 6218.86 Da); 29.4 min, *m*/*z* of 1303.993 (5+ charges, peptide mass 6514.93 Da), and 46.8 min, *m*/*z* of 918.745 (4+ charges, peptide mass 3670.95 Da).

Using the new HRS system, a large binding peak eluting at 32.3 min was found in the *Pseudonaja affinis* venom (Figure 4). After correlation with the parallel MS chromatogram, it was found that this bioactive shows an ion with *m*/*z* value of 1244.779 (5+ charges, peptide mass 6218.86 Da). There were two additional ligands found in the venom that showed a low binding peak, eluting at 29.4 and 46.8 min with *m*/*z* values of 1303.993 (5+ charges, peptide mass 6514.93 Da) and 918.745 (4+ charges, peptide mass 3670.95 Da), respectively.

**Figure 5:**
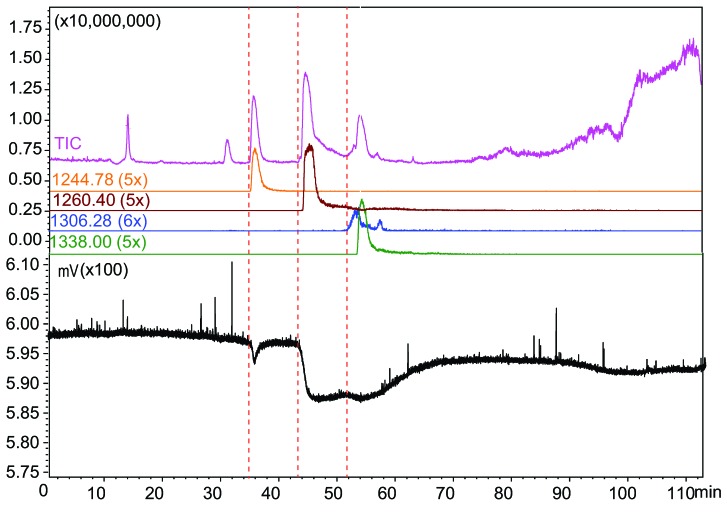
Microfluidic on-line HRS screening of *Pseudonaja inframacula*. The identified ligands: 35.1 min, *m*/*z* of 1244.779 (5 times charged, 6218.86 Da); 43.4 min, *m*/*z* value of 1260.397 (5+ charges, peptide mass 6296.95 Da); 52.0 min, *m*/*z* value of 1306.287 (5+ charges, peptide mass 7831.68 Da) or to 1338.006 (5+ charges, peptide mass 6684.99 Da).

In the venom of *Pseudonaja inframacula*, two large binding and one small binding ligand were found with the on-line HRS screening system (Figure 5). A ligand with a low binding peak eluting at 35.1 min shows an ion with *m*/*z* value of 1244.779 (5+ charges, peptide mass 6218.86 Da). The second bioactive eluting at 43.4 min shows an ion with *m*/*z* value of 1260.397 (5+ charges, peptide mass 6296.95 Da). The third ligand eluting at 52.0 min and could correspond to a venom protein with *m*/*z* values of 1306.287 (5+ charges, peptide mass 7831.68 Da) or to the *m*/*z* value of 1338.006 (5+ charges, peptide mass 6684.99 Da). The mass of the protein with *m*/*z* value of 1306.287 (peptide mass 7831.68 Da) was correlated to the molecular mass of the known toxin pseudonajatoxin-b previously identified from *Pseudonaja textilis*, which is known to bind to the α7-nAChR [27].

**Figure 6:**
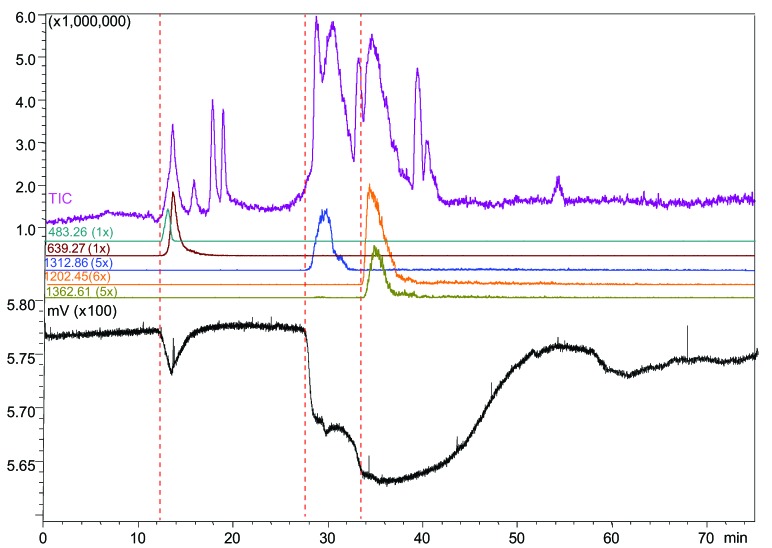
Microfluidic on-line HRS of *Dendroapsis polylepis* venom. The identified ligands: 13.0 min, *m*/*z* value of 639.271 or 483.255 (1+ charge, peptide masses 638.26 and 482.25 Da); 29.2 min, *m*/*z* of 1312.864 (5+ charges, peptide mass 6559.28 Da); 33.6 min, *m*/*z* 1202.608 (6+ charges, peptide mass 7209.60 Da) or *m*/*z* 1362.613 (5+ charges, peptide mass 6808.03 Da).

In *Dendroapsis polylepis* venom, we found a bioactive with a small binding peak eluting at 13.0 min, which was correlated to two co-eluting compounds with a *m*/*z* value of 639.271 or 483.255 (1+ charge) (Figure 6). Two bioactives showing large binding peaks were detected at 29.2 min corresponding to a *m*/*z* of 1312.864 (5+ charges, peptide mass 6559.28 Da), and at 33.6 min corresponding to two co-eluting proteins with a *m*/*z* of 1202.608 (6+ charges, peptide mass 7209.60 Da) and *m*/*z* of 1362.613 (5+ charges, peptide mass 6808.03 Da).

A list of all identified *m*/*z* values of the bioactives found, with charge state and calculated molecular mass, is shown in Supplementary Information Table S2.

### 2.5. Protein Identification

To study the feasibility of identifying a bioactive compound detected by the HRS system, we performed in-solution tryptic digestion of the bioactive with an *m*/*z* value of 1244.78 from the *Pseudonaja affinis*. We first refractionated the venom using a conventional bore LC system from which the effluent was post-column split with 10% being analyzed by MS for detecting the compound with *m*/*z* of 1244.78, and 90% being directed to a fraction collector. The fraction with the protein of interest was collected and subjected to tryptic digestion for proteomics analysis by nanoLC-MS (see Experimental section). The MS and MS/MS data obtained were subjected to a Mascot search using the Uniprot.org database which indicated the bioactive to be the Short neurotoxin 3 from *Pseudonaja textilis* (Uniprot number Q9W7K0) with a sequence coverage of 93% and Mascot score of 832 (Supplementary Information Figure S2).

## 3. Experimental Section

### 3.1. Chemicals and Biological Reagents

DAHBA ((E)-3-(3-(4-diethylamino-2-hydroxybenzylidene)-3,4,5,6-tetrahydropyridin-2-yl)pyridine), VUF11234 ((E)-1-methyl-5-((2-(pyridin-3-yl)-5,6-dihydropyridin-3(4H)-ylidene)methyl)indoline and VUF10907 ((E)-3-(3-(4-Dimethylaminobenzylidene)-3,4,5,6-tetrahydropyridin-2-yl)pyridine) were synthesized in house as described by Kool *et al*. [25]. VUF10166 was synthetized as described by Thompson *et al*. [28]. ELISA blocking reagent was purchased from Hoffmann-La Roche (Mannheim, Germany). The ULC-MS grade 99.97% acetonitrile (ACN) and 99.95% trifluoroacetic acid (TFA) were obtained from Biosolve (Valkenswaard, The Netherlands). HPLC grade water was produced by a Milli-Q purification system from Millipore (Amsterdam, The Netherlands). NaCl, dithiothreitol (DTT), iodoacetamide (IAM), trypsin from bovine pancreas, Trizma base, KH_2_PO_4_, Na_2_HPO_4_, NH_4_HCO_3_, 5-fluorotrypamine and quipazine were purchased from Sigma-Aldrich (Zwijndrecht, The Netherlands). Granisetron, tropisetron, serotonine-HCl, RS56812, mirtazapine, SR57227, zacopride, iodophenpropit, B-HT920, RS16566 and palonosetron were obtained from Tocris Bioscience—R&D Systems Europe (Abington, Oxon, UK).

### 3.2. Expression and Purification of 5HTBPs

A1B2D1_R_ and A1B2D1_W_ 5HTBP mutants were expressed and purified as described by Kesters *et al.* [10]. In short, the mutagenesis was induced using a QuikChange-based strategy on Ac-AChBP (from snail species *Aplysia californica*). Protein was expressed using a Bac-to-Bac expression system. The protein was purified by affinity chromatography with nickel Sepharose as affinity material (GE Healthcare, Eindhoven, The Netherlands) followed by size exclusion chromatography (Superdex 200 column, GE Healthcare, Eindhoven, The Netherlands) using a 20 mM Tris buffer of pH 8.0 containing 150 mM NaCl as eluent. Fractions corresponding to pentameric protein were pooled, concentrated to 6 mg/mL and stored at −80 °C until use.

### 3.3. Snake Venom Samples

Lyophilized venoms from Dendroapsis polylepis, Pseudonaja affinis and Pseudonaja inframacula, Crotalus adamanteus, Crotalus horridus atricaudatus, Bitis arietans and Bitis nasicornis were acquired, freeze-dried and send to us by Ryan Mccleary and Prof. R.M. Kini (National University Singapore). The venom samples (10 mg/mL) were dissolved in Milli-Q water/ACN/TFA 95:5:0.1%. After analysis, the remainder of samples was stored at −20 °C for further use, if needed.

### 3.4. Fluorescence Enhancement by Anabaseine Derivatives with 5HTBP Constructs A1B2D1_R_ and A1B2D1_W_

The fluorescence enhancement properties of three anabaseine derivatives (Supplementary Information Figure S3) bound to the orthosteric binding pocket of two different 5HTBP variants were measured using 1 cm wide glass cuvettes in a Perkin-Elmer LS50B fluorometer (Groningen, The Netherlands). The excitation spectra, λmax (nm), and ε (L·mol**^−^**^1^·cm**^−^**^1^) values of the anabaseine derivatives are described by Kool *et al*. [25]. The emission spectra from 500–595 nm were recorded with 0.5 nm intervals with a fixed 488 nm excitation.

### 3.5. Fluorescence Enhancement Microplate Reader Assay

The buffer used for development of the 5HTBP fluorescence enhancement assay consisted of 1 mM KH_2_PO_4_, 3 mM Na_2_HPO_4_, 0.16 mM NaCl, 20 mM trizma base/HCl at pH 7.5 with addition of 400 μg/mL ELISA BR [21]. The fluorescence enhancement was measured with a Victor3 Microplate reader from Perkin-Elmer. The excitation and emission wavelengths were set to 485 and 520 nm, respectively. Black-bottomed 96-well microplates were purchased from Greiner Bio-One (Alphen aan den Rijn, The Netherlands). The final assay volume was 100 µL per well. The 5HTBP was first mixed with the ligands, and then with the tracer ligand. The assay mixture was incubated for 5 min before the measurement. The pharmacological validation was performed by measurement of IC_50_ curves of twelve 5-HT_3_R ligands and comparing the results obtained with the results obtained from a radioligand binding assay (see below). The z’ factors for the determination of assay quality were calculated according to Zhang [29].

### 3.6. Radioligand Binding Assay with [^3^H]-granisetron

The competitive radioligand-binding assay was performed as described by Kesters *et al.* 2013 [10]. His-tagged A1B2D1_R_ 5HTBP was diluted in buffer (10 mM HEPES/0.05% Tween) to obtain 50 ng binding protein per well. Serial dilutions of the ligands (10**^−^**^3^ to 10**^−^**^12^ M from stock concentrations of 10 or 100 mM in DMSO) were used for IC50 determinations. The concentration of the radioligand [3H]-granisetron (Perkin-Elmer, Life Science, Groningen, The Netherlands, specific activity ~85 Ci/mmol) was 1.99 nM. After addition of PVT Copper His-Tag SPA beads (final concentration 2 mg/mL), the final well volume was 100 µL. The plates (white Optiplates, Perkin-Elmer Life Science, Groningen, The Netherlands) were incubated at room temperature under continuous shaking while protected from light for 1.5 h. The SPA beads were then allowed to settle for 3 h in the absence of light before counting in a Wallac 1450 MicroBeta (Perkin-Elmer Life Science, Groningen, The Netherlands). All radioligand binding data were fit by a non-linear, least squares curve analysis procedure using Graphpad Prism (version 5, GraphPad Software).

### 3.7. Microfluidic On-Line Assay Optimization in Nano-LC Flow-Injection Mode

The optimal concentrations of the tracer ligand and the binding protein used in the microfluidic on-line assay were first evaluated in nano-LC flow injection mode (without using an analytical column) in order to measure the pure compounds without chromatographic retention. The schematic overview of the microfluidic on-line assay in nano-LC flow-injection mode is shown in Supplementary Information Figure S4A. An Ultimate 3000 nano-LC system (Thermo Scientific, Breda, The Netherlands) was used in combination with the miniaturized bioaffinity detection system. Sample injection volumes were 500 nL. Bioaffinity was measured with a microfluidic fluorescence detection system [30] in which the eluent flow from the nano-LC instrument was first incubated in an on-line 4-µL volume microfluidic chip with the assay mixture consisting of 5HTBP and the fluorescent tracer DAHBA in assay buffer. The assay mixture was continuously fed to the microfluidic system at a flow rate of 5 µL/min using a syringe pump positioned in the dark at 4 °C. Infusion was possible for up to 8 h before refilling of the syringe was needed. After on-line incubation, the fluorescence was measured with a LED-induced fluorescence detector [30]. After assay optimization, the pharmacological validation of the on-line assay was performed by measuring IC_50_ curves with known 5-HT_3_R ligands. Calculation of the final concentration of ligands in the assay, which is lower than the injection volume due to dilution by the chromatographic separation and in the microfluidic assay, was performed as described elsewhere [26,31].

### 3.8. Microfluidic On-Line HRS with Snake Venoms

The microfluidic on-line HRS system was similar as described in [21] (Supplementary information Figure S4B). In this system, the nano-LC eluate was split post-column in a 1:1 ratio. One part was directed to the microfluidic fluorescence detection system, and the other part to an ion-trap-time-of-flight (IT-TOF) mass spectrometer equipped with a Picoview nano-Electrospray ionization source (Shimadzu, 's-Hertogenbosch, The Netherlands). The MS was operated in positive-ion mode with an interface voltage of 1.7 kV, and a heating block and curved desolvation line temperature of 200 °C. For chromatographic separation of the snake venom proteins, a 150 mm × 75 µm internal diameter capillary column was packed in-house with Aqua C18 particles (5 µm, 200 Å; Phenomenex, Utrecht, The Netherlands). For gradient elution mobile phase eluent A consisted of water/ACN/TFA 99/1/0.1% and eluent B of water/ACN/TFA 1/99/0.1%. The snake venoms were separated with two nano-LC gradient programs and screened using miniaturized on-line HRS. First, a relatively fast gradient program of 75 min was used: 0–5 min isocratic 5% B; 5–10 min, linear 5%–15% B; 10–40 min, linear 15%–40% B; 40–50 min, linear 50%–70% B; 50–60 min, isocratic 70%; 60–75 min, re-equilibration 5% B. When needed, reanalysis of a venom was performed using a 115-min gradient: 0-5 min, isocratic 5% B; 5–15 min, linear 5%–15% B; 15–75 min, linear 15%–45%; 75–85 min, linear 45%–70% B; 85–100 min, isocratic 70% B; 100-115 min, re-equilibration 5% B. For screening, 500 nL of each snake venom (10 mg/mL) was injected.

### 3.9. Purification of Bioactives from Snake Venoms

The purification of a bioactive from the *Pseudonaja affinis* snake venom was performed by using a normal bore LC-MS setup with a 10/90% split to MS analysis and fractionation, respectively. The system details are described elsewhere [32]. In short, the eluents for the LC gradient were pumped with two Shimadzu LC-2AD HLPC pumps at 0.6 mL/min. Mobile phase eluent A consisted of water/ACN/FA 98/2/0.1% and eluent B of water/ACN/FA 2/98/0.1%. For chromatographic separation a C18 column (XBridge, 100 mm × 4.6 mm, 3.5 µm particle size, Waters, Milford, MA, USA) and a linear gradient from 0–90% B in 20 min was used. The column effluent (90%) was fractionated using a GILSON 235P Autoinjector modified to operate as fraction collector, while 10% of the effluent was directed to a Micromass Q-TOF Ultima mass spectrometer (Waters, Milford, MA, USA). MS measurement was in positive ion-mode using a 150 °C source temperature, 200 °C desolvation temperature, and 3 kV capillary voltage. Fractions were collected at a rate of 6 s/well into a black 384-well microplate (Greiner Bio One, Alphen aan den Rijn, The Netherlands). The microplate was freeze-dried and stored at −20 °C until further use. Only the fractions of interest were re-dissolved for further analysis.

### 3.10. Tryptic Digestion

The freeze-dried fraction of interest was dissolved in 18 µL 25 mM NH_4_HCO_3_, pH 8 digestion buffer. Present protein was reduced with 2 µL 50 mM dithiothreitol (DTT) at 50 °C for 30 min. After reduction, 7 µL digestion buffer was added, and 3 µL 100 mM iodoacetamide (IAM) was added for the alkylation. Finally, 1 μL of 0.1 µg/µL trypsin was added and incubated for 2 h at 37 °C. After this incubation, a second aliquot of 1 μL 0.1 µg/µL trypsin was added and the solution was left at 30 °C overnight for digestion. The next day, the digestion was stopped by addition of 1% formic acid (final concentration).

The tryptic digests were analyzed by a micrOTOF-Q mass spectrometer (Bruker Daltonics, Bremen, Germany) equipped with an electrospray nano ion source and an Ultimate-3000 nano-LC system (Thermo Fisher Scientific). A 5-mm Pepmap 100 C18 pre-column (300 µm ID, 5 µm particle size, Thermo Fisher Scientific) was used to trap the peptides. The separation was performed on a Acclaim Pepmap RSLC analytical column (75 µm × 15 cm, Thermo Fisher Scientific). Eluent A consisted of water/FA 100/0.1% and eluent B of water/ACN/FA 20/80/0.1%. The sample was loaded on the pre-column with 15 μL/min of water/ACN/TFA 98/2/:0.1% for 3 min. After loading, the sample was separated at a flow rate of 600 nL/min applying a 0–30 min linear gradient from 4% to 55% B, and between 30–36 min a linear gradient from 55% to 90% B. Subsequently, 1 min isocratic at 90% B was followed by column re-equilibration at 4% B for 10 min. The MS analysis was performed in positive-ion mode (*m*/*z* 50–2000) using 1.5 L/min drying gas, 150 °C dry heater, 2.5 kV capillary voltage. Data dependent MS^2^ was performed on the three most abundant ions in the recorded full MS spectra using 25 eV collision energy. The MS data were processed and analyzed with Mascot Distiller (version 2.3.2,) and Mascot server (version 2.2) from Matrix Science (London, UK).

## 4. Conclusions

In this work, two new fluorescence-based assays are described, which can be used to find new ligands of the 5-HT_3_ receptor and nACh receptor from pure compound libraries and mixture libraries. Both assays use an engineered binding protein, which has the scaffold of the AChBP and the ligand recognition properties of the 5-HT_3_ receptor in the binding pocket. The assays were optimized and validated in a micro-plate reader and subsequently transferred to a microfluidic on-line assay format capable of screening mixtures. The assays were found to be robust and well correlating with IC_50_s measured using a conventional radioligand binding assay.

The main strength of a fluorescence-based bioassay like the assay developed is the cost-effectiveness compared to the radioligand binding assays and the ability to perform in flow-injection mode, which can be coupled post-column to mass spectrometry. These assays are well suitable for mixture analysis. As an application example for the microfluidic on-line assay the screening of several snake venoms for identification of new peptides binding to the 5HTBP was demonstrated. The venoms from *Pseudonaja inframacula*, *Pseudonaja affinis* and *Dendroaspis polylepis* were screened and we successfully pinpointed the accurate masses of bioactives acting on the 5HTBP. The complete workflow of protein identification of bioactive proteins was finally demonstrated with one of bioactives from *Pseudonaja affinis* found during the screening process. Using the accurate mass identified with the microfluidic on-line screening, the venom was subsequently fractionated and the fraction containing the protein of interest was digested with trypsin prior to proteomics based identification of the protein. The Mascot search identified this protein to be Short neurotoxin 3 from *Pseudonaja textilis* (Uniprot number Q9W7K0). In the future these screening assays can be used for screening campaigns to identify novel bioactive compounds from complex mixtures.

## Supplementary Materials

Supplementary materials can be accessed at: http://www.mdpi.com/2072-6651/7/7/2336/s1.
