# Supplementary material for: Development of Plate Reader and On-Line Microfluidic Screening to Identify Ligands of the 5-Hydroxytryptamine Binding Protein in Venoms
Source: Toxins (Basel). 2015 Jun 24;7(7):2336–53. doi: 10.3390/toxins7072336 (PMC4516916; doi:10.3390/toxins7072336)
Supplement: Supplementary file 1 [file toxins-07-02336-s001.pdf]

Supplementary Information

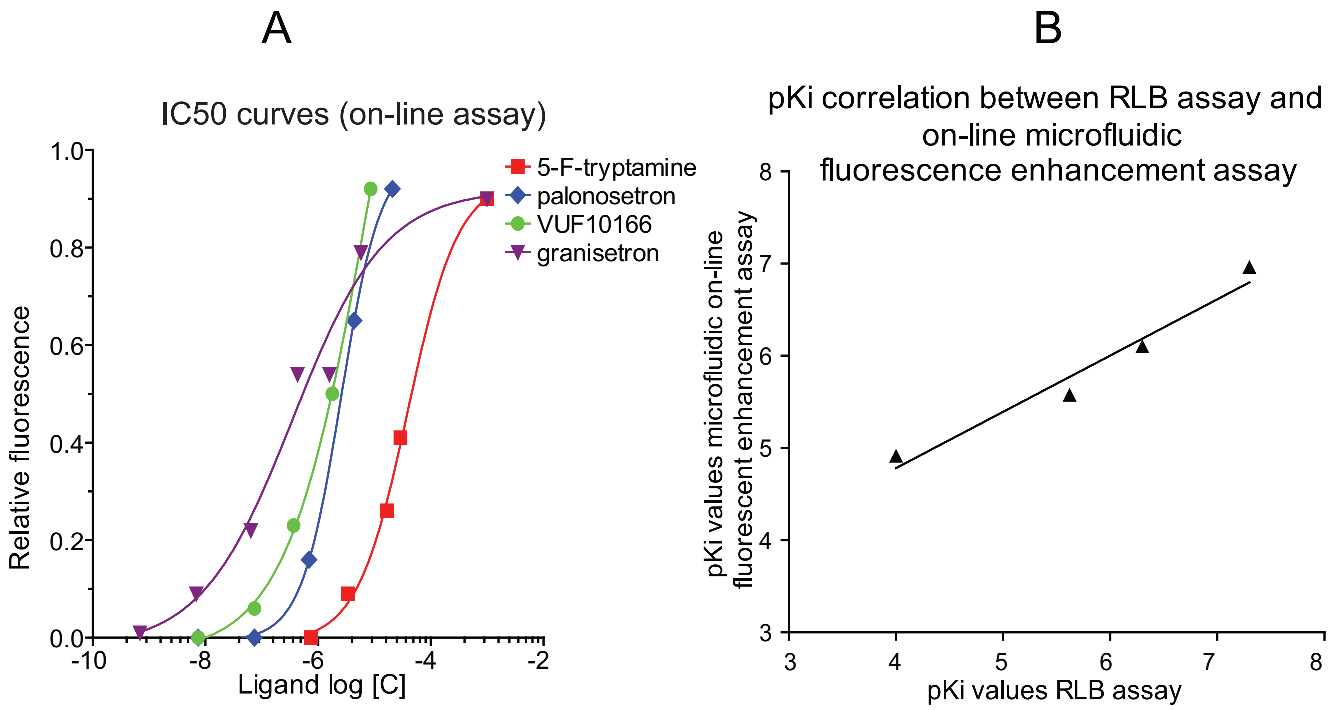

Figure S1. (**A**) Concentration-response of four 5HT3 receptor ligands obtained with the microfluidic on-line system. Due to dilution effects in the nano-LC and on-line assay, the actual concentration in the assay is lower than the injected concentration. The dilution factor of ligands injected was calculated as described by Falck *et al*. (Falck, de Vlieger *et al*. 2010), and then used to estimate the assay concentration of the analyzed ligand; (**B**) Correlation between the RLB assay and the microfluidic on-line fluorescence enhancement assay ($r^2 = 0.9598$).

Mascot score: 832
Short neurotoxin 3 OS=Pseudonaja textilis GN=SNTX3 PE=2 SV=1 (Uniprot number: Q9W7K0)
Sequence coverage: 67%

1 MKTLLLTLVM VTIMCLDLGY TLTCYKGYHD TVVCKPHETI CYRYLVPATH
51 GNAIPARGCG TSCPGGNHPV CCSTDLCNK

Sequence coverage without the signal peptide (MKTLLLTLVMVTIMCLDLGYT): 93 %

1 LTCYKGYHD TVVCKPHETI CYRYLVPATH GNAIPARGCG TSCPGGNHPV CCSTDLCNK

MS/MS fragmentation:

GCGTSCPGGNHPVCCSTDLCNK

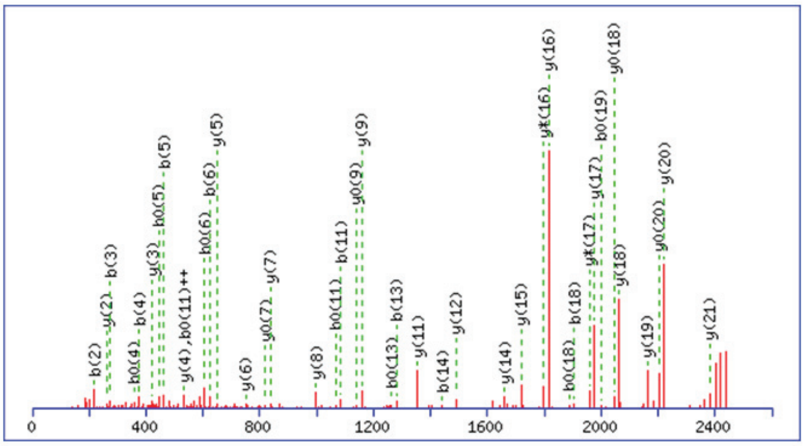

GYHDTVVCKPHETICYR

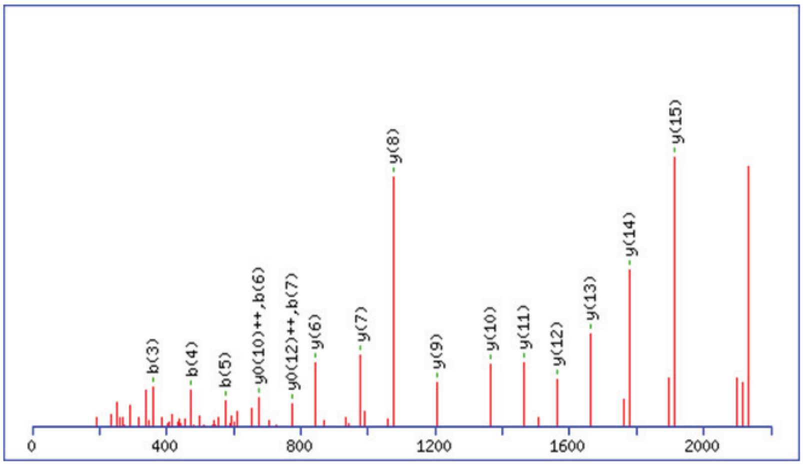

YLVPATHGNAIPAR

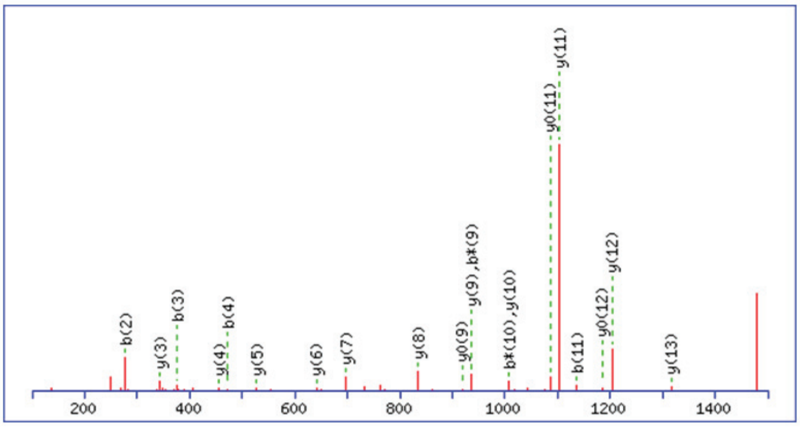

Figure S2. Mascot results from the tryptic digestion of the bioactive with *m*/*z* value of 1244.78 from *Pseudonaja affinis*.

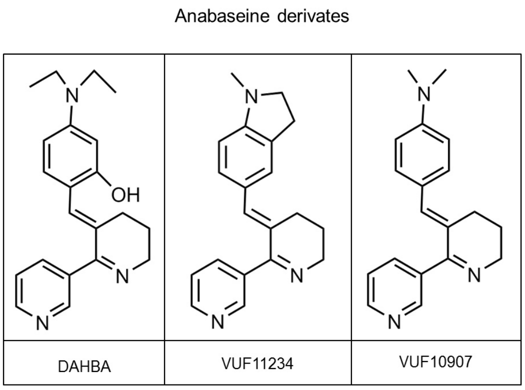

Figure S3. Structure of the three anabaseine derivates tested in the study.

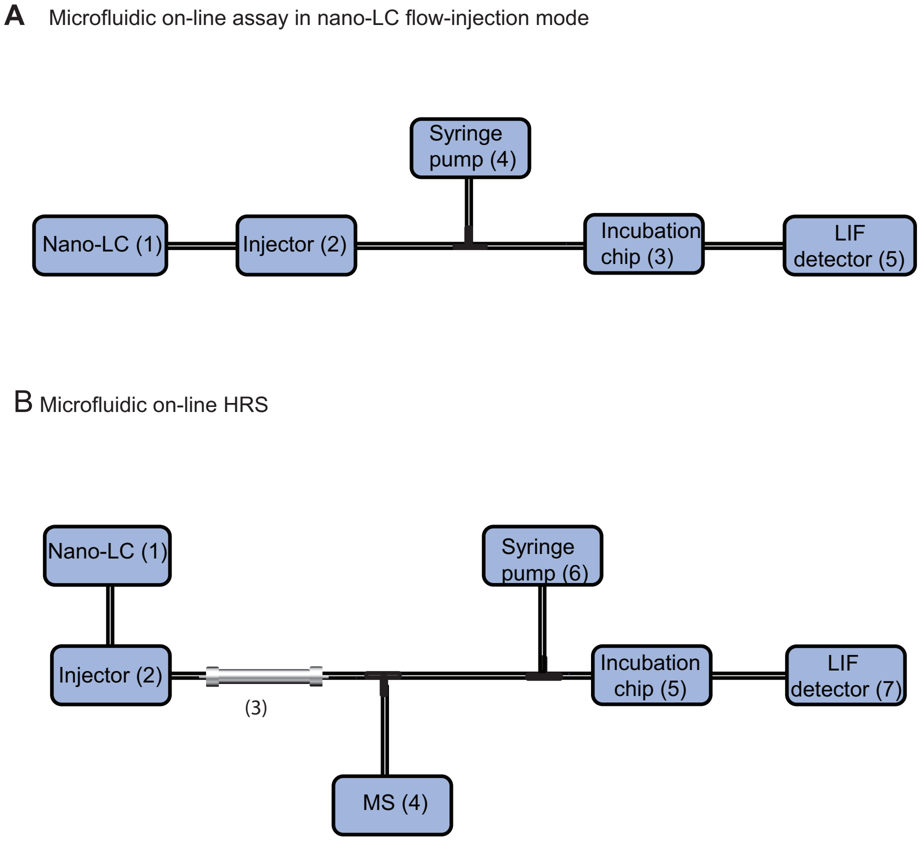

Figure S4. (**A**) Schematic view of the microfluidic on-line assay in nano-LC flow-injection mode. With a nano-LC system (1) 500 nL of samples are injected (2). The eluent flow is directed into a 4 µl microfluidic incubation chip (3) where the sample is mixed with the bioassay mixture, infused in the chip by a syringe pump (4). The fluorescence signal was detected by an in-house built LED-induced fluorescence detector (5); (**B**) Schematic view of the microfluidic on-line HRS setup. 500 nL of samples are injected (2) and separated with nano-LC (1). After separation by the capillary column (3) the effluent flow was spit in 1:1 ratio. One part of the flow was directed to a high resolution MS (4), and the other part of the flow was directed into a 4 µl microfluidic incubation chip (5) where it was mixed with the bioassay mixture infused by a syringe pump (6). After incubation the fluorescence signal was detected by an in-house built LED-induced fluorescence detector (7).

Table S1. Comparison of Ki values measured for 14 5HT-3 ligands using the fluorescence enhancement plate reader assay and the radioligand binding assay.

**Ligands**	**Ki fluorescence enhancement (A1B2D1R 5HTBP mutant) (μM)**	**Ki RLB (A1B2D1R 5HTBP mutant) (μM)**
granisetron	0.24 ± 0.14	0.05 ± 0.01
serotonine	103.03 ± 32.01	218.27 ± 24.98
tropisetron	0.04 ± 0.72	0.01 ± 0
quipazine	72.98 ± 32.22	53.84 ± 13.74
VUF10166	11.54 ± 3.58	2.39 ± 0.58
RS56812	3.57 ± 0.91	1.6 ± 0.34
mirtazapine	12.82 ± 3.99	9.29 ± 1.87
SR57227	85.03 ± 7.62	39.74 ± 15
zacopride	4.05 ± 1.14	1.62 ± 0.57
iodophenpropit	1.44 ± 0.2	0.23 ± 0.06
B-HT920	3.85 ± 0.76	0.93 ± 0.24
RS 16566	3 ± 0.02	0.38 ± 0.09
5-fluorotryptamine HCL	110.35 ± 14.03	100.95 ± 50.19
palonosetron	2.2 ± 0.63	0.51 ± 0.11

Table S2. Mass of bioactives binding to 5THBP found in snake venoms.

**Species**	**Most abundant *m*/*z***	**Charge state**	**~Nominal mass (Da)**
Pseudonaja affinis	1244.779	5	6218.86
Pseudonaja affinis	1303.993	5	6514.93
Pseudonaja affinis	918.745	4	3670.95
Pseudonaja inframacula	1244.779	5	6218.86
Pseudonaja inframacula	1260.397	5	6296.95
Pseudonaja inframacula	1306.287	6	7831.68
Pseudonaja inframacula	1338.006	5	6684.99
Dendroapsis polylepis	639.271	1	638.26
Dendroapsis polylepis	483.255	1	482.25
Dendroapsis polylepis	1312.864	5	6559.28
Dendroapsis polylepis	1362.613	5	6808.03
Dendroapsis polylepis	1202.608	6	7209.60
